# Correlation between heart rate variability and pulmonary function adjusted by confounding factors in healthy adults

**DOI:** 10.1590/1414-431X20154435

**Published:** 2016-02-02

**Authors:** M.S. Bianchim, E.F. Sperandio, G.S. Martinhão, A.C. Matheus, V.T. Lauria, R.P. da Silva, R.C. Spadari, A.R.T. Gagliardi, R.L. Arantes, M. Romiti, V.Z. Dourado

**Affiliations:** 1 Laboratório de Epidemiologia e Movimento Humano, Departamento de Ciências do Movimento Humano, Universidade Federal de São Paulo, Santos, SP, Brasil; 2 Departamento de Biociências, Universidade Federal de São Paulo, Santos, SP, Brasil; 3 AngioCorpore Instituto de Medicina Cardiovascular, Santos, SP, Brasil

**Keywords:** Autonomic nervous system, Spirometry, Smoking

## Abstract

The autonomic nervous system maintains homeostasis, which is the state of balance in the body. That balance can be determined simply and noninvasively by evaluating heart rate variability (HRV). However, independently of autonomic control of the heart, HRV can be influenced by other factors, such as respiratory parameters. Little is known about the relationship between HRV and spirometric indices. In this study, our objective was to determine whether HRV correlates with spirometric indices in adults without cardiopulmonary disease, considering the main confounders (e.g., smoking and physical inactivity). In a sample of 119 asymptomatic adults (age 20-80 years), we evaluated forced vital capacity (FVC) and forced expiratory volume in 1 s (FEV_1_). We evaluated resting HRV indices within a 5-min window in the middle of a 10-min recording period, thereafter analyzing time and frequency domains. To evaluate daily physical activity, we instructed participants to use a triaxial accelerometer for 7 days. Physical inactivity was defined as <150 min/week of moderate to intense physical activity. We found that FVC and FEV_1_, respectively, correlated significantly with the following aspects of the RR interval: standard deviation of the RR intervals (*r* =0.31 and 0.35), low-frequency component (*r* =0.38 and 0.40), and Poincaré plot SD2 (*r* =0.34 and 0.36). Multivariate regression analysis, adjusted for age, sex, smoking, physical inactivity, and cardiovascular risk, identified the SD2 and dyslipidemia as independent predictors of FVC and FEV_1_ (*R* ^2^=0.125 and 0.180, respectively, for both). We conclude that pulmonary function is influenced by autonomic control of cardiovascular function, independently of the main confounders.

## Introduction

The autonomic nervous system maintains visceral functions through the activity of its sympathetic and parasympathetic branches. At times, the two branches operate in an antagonistic manner, generating a dynamic balance known as autonomic control. Analysis of heart rate variability (HRV) is a noninvasive and simple method for assessing autonomic control of the heart. The oscillation in the interval between consecutive heartbeats is an indicator of the integrity of the cardiovascular system and its ability to adapt to environmental changes (1).

Decreased HRV is associated with increased risk of morbidity and mortality after acute myocardial infarction (2). In addition, autonomic imbalance has been linked to the development of a wide range of diseases, including arteriosclerosis, congestive heart failure, diabetic neuropathy, obesity, depression, and stress (3 4 5).

The respiratory cycle also affects autonomic control. The heart rate increases during inspiration and decreases during expiration, causing fluctuations in HRV (6). This physiological phenomenon is known as respiratory sinus arrhythmia (RSA). There are several ways to measure RSA, the most common being through analysis of the high-frequency (HF) component of HRV (7). The spectral variable HF is also referred to as RSA or respiratory rate because it has the same range as typical healthy adult respiration (7). This indicates that there is functional synchrony between the heart and lungs. However, the relationship between HRV and pulmonary function is unclear.

Many factors influence pulmonary and cardiac function. Pulmonary function is influenced by lifestyle and cardiovascular risk factors such as obesity, high blood pressure, high cholesterol, metabolic syndrome, physical inactivity, and smoking (8 9 10 11 12 13 14). Dyslipidemia and elevated heart rate are independent risk factors for pulmonary function impairment (15). In addition, pulmonary function is inversely associated with levels of inflammation-sensitive plasma proteins (16). The role of lipids in inflammatory processes is well known and might explain the role of dyslipidemia in the development of pulmonary diseases (16). Physical activity also plays an important role in regulating cardiac and pulmonary function. Furthermore, regular physical activity increases HRV by increasing parasympathetic activity at rest. Moderate to vigorous physical activity can reduce the decline in pulmonary function among smokers, preventing the development of chronic obstructive pulmonary disease (COPD) as well as reducing mortality (17). Nevertheless, longtime smokers present lung tissue remodeling and a pronounced decline in pulmonary function with aging. Moreover, HRV is compromised in smokers (18). Those effects are attributable to increased release and reduced catabolism of catecholamines, together with decreased vagal tone (18). Increased activation of the sympathetic nervous system in smokers has an important clinical role, as has been widely reported (18 19 20).

Although the correlation between HRV and pulmonary function has been investigated in respiratory diseases such as COPD and asthma (21,22), few studies have evaluated that correlation in healthy adults (23). We hypothesized that lower HRV indices are associated with impaired pulmonary function, independently of factors such as smoking, level of physical activity in daily life, and cardiovascular risk. Therefore, the objective of the present study was to determine whether HRV correlates with the main spirometric indices in asymptomatic adults, as well as whether those correlations remain significant after being adjusted for the main confounders.

## Material and Methods

The Epidemiological Study of Human Movement and Hypokinetic Diseases is a longitudinal, population-based cohort study investigating whether sedentary behavior and physical inactivity are associated with the occurrence of hypokinetic diseases, especially cardiorespiratory diseases. From those participating in this ongoing study, we recruited 119 participants (50 men and 69 women) who were asymptomatic and free of cardiorespiratory disease. We excluded individuals with Chagas disease, acute myocardial infarction, coronary heart disease, COPD, uncontrolled hypertension, diabetes, evidence of osteoarticular problems, or a recent history of respiratory infection, as well as those with a high risk of cardiac disease and those using cardiovascular drugs (e.g., beta-adrenoceptor antagonists). The study was approved by the Ethics Committee for Research in Humans, Universidade Federal de São Paulo (Protocol #186.796), and all participants provided their written informed consent.

### Health evaluation

During clinical evaluation of participants, we collected data related to level of education and medication use. In addition, we collected detailed information on the following cardiovascular risk factors: age, obesity, family history of cardiovascular disease, hypertension, dyslipidemia, sedentary lifestyle, angina (stable or unstable), dizziness, and syncope. Dyslipidemia was defined as total cholesterol or triglyceride levels higher than 240 mg/dL. Body mass index (BMI) was calculated after measuring weight and height on a scale equipped with a stadiometer (2124; Toledo, Brazil). Participants with BMI ≥30 kg/m^2^ were considered obese (24). Cardiovascular risk was classified as mild or moderate according to the number of risk factors (<2 or ≥2, respectively). Participants who reported current smoking and having smoked at least 100 cigarettes in their lifetime were classified as smokers (25,26). Smoking history was calculated in pack-years. Participants were asked about their history of COPD and asthma, as well as about exposure to dusty environments and chemicals within the last year.

### Pulmonary function

We performed pulmonary function tests with a spirometer (Quark PFT; Cosmed, Italy) using the forced vital capacity (FVC) maneuver. The maneuver could be repeated up to seven times until three results were reproducible. The turbines were calibrated before each test. Forced expiratory volume in 1 s (FEV_1_), FVC, and the FEV_1_/FVC ratio were determined. Spirometric indices are expressed as absolute values and as percentages of the predicted values (27).

### HRV

For each participant, we measured RR intervals using a heart rate monitor (Polar RS800; Polar Electro Oy, Finland), while the participant was at rest in the supine position. Although intervals were monitored for 10 min, our analysis included only the data obtained within a 5-min window in the middle of the monitoring period, the initial and final 150-s periods being excluded. The data were then transferred to a computer and stored using compatible software (Polar ProTrainer 5; Polar Electro Oy). The data were visually inspected, and any inappropriate or premature beats were corrected by interpolation. Those RR intervals showing a >20% difference in relation to the adjacent intervals were filtered (1). The results were then stored in text files and transferred using Kubios HRV version 2.2 software (University of Eastern Finland: http://kubios.uef.fi/KubiosHRV/Download/). The linear indices obtained in the time domain were as follows: the mean RR interval, the root mean square of successive differences between adjacent normal RR intervals, the standard deviation of the RR intervals, the number of adjacent normal RR intervals differing by >50 ms, and the proportion of adjacent normal RR intervals differing by >50 ms. In the frequency domain, we obtained the following linear indices: the HF component, the low-frequency (LF) component, and the LF/HF ratio. The geometric indices assessed were short-term variability (SD1) and long-term variability (SD2) of the Poincaré plot (4). We also analyzed the nonlinear indices α1 and α2 (28).

HRV can be influenced by various conditions including blood pressure, anxiety, left ventricular ejection fraction, lung volume, breathing pattern, respiratory frequency, and medication use. To minimize such interference, all analyses were performed at the same time of day, with participants at rest in the supine position. Participants were instructed to avoid drinking coffee, tea, soft drinks, and alcoholic beverages, as well as to avoid engaging in physical activities and avoid smoking before the HRV test. All participants remained at rest for 5 min before the test. They were instructed to breathe normally and avoid speaking during the test.

### Physical activity in daily life

The level of physical activity in daily life was measured with a triaxial accelerometer (GT3X+; Actigraph, USA). Each instrument was programmed according to the characteristics of the participant (sex, age, dominant side, height, and body mass). Participants were instructed to wear the accelerometer at the waist above the dominant hip for 7 days. They were instructed to remove the accelerometer during sleeping and water activities, including bathing. Only days with at least 12 h of continuous monitoring were considered valid. Energy expenditure was measured and physical activity was classified as mild, moderate, vigorous, or very vigorous (29). Participants who were unable to engage in moderate to vigorous physical activity for at least 150 min/week were considered physically inactive. Therefore, physical inactivity was analyzed as a categorical variable.

### Statistical analysis

Sample size was calculated based on the number of predictors in the multiple regression models, as follows: age, sex, BMI, HRV, smoking, physical inactivity, and cardiovascular risk factors (family history of cardiovascular disease, hypertension, dyslipidemia, angina, and syncope). Considering a correction coefficient (*r* ) of 0.80 and a coefficient of determination (*R* ^2^) of 0.64, with 11 predictors, the minimum sample size required for this study would be 110 participants. In multivariate linear regressions, spirometric indices were analyzed as outcomes.

Statistical analysis was performed using the IBM SPSS Statistics, Version 23.0 (IBM Corp., USA). Data were analyzed using descriptive statistics. We used the Kolmogorov-Smirnov to assess data normality. Continuous variables are presented as mean±standard deviation or as median (interquartile range), depending on the distribution of the data (symmetrical or asymmetrical). Categorical variables are presented as frequencies. The Pearson or Spearman correlation coefficient was used to evaluate the correlations, also depending on the distribution of the data. Stepwise multiple linear regressions were used to identify correlations between HRV indices as predictors and spirometric indices as outcomes. The HRV indices were divided into time, frequency, and nonlinear domains. Those HRV indices that presented the strongest correlations with FEV_1_ and FVC in each category were selected as predictors for inclusion in the regression models. The multiple regression models were adjusted for the main confounders, such as smoking and cardiovascular risk including physical inactivity assessed directly by triaxial accelerometry. We also adjusted the models for the use of medications other than cardiovascular drugs. The probability of a type I error was set at 5%.

## Results

Participants were, on average, middle-aged adults, ranging in age from 20 to 80 years (Table 1). The mean BMI was 27±5 kg/m^2^ (within the range of overweight), and 31 (26.1%) of the 119 participants were obese. According to the spirometric indices, the participants were free of respiratory disturbances. However, nine participants (7.6%) had arterial hypertension, seven (5.9%) had diabetes, and 24 (20.2%) had dyslipidemia. In addition, 19 (16%) were smokers and 14 (11.9%) were physically inactive.

**Table:**
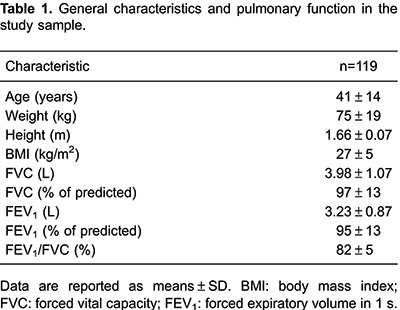

We found that HRV correlated moderately, but significantly, with the spirometric indices (Table 2). The indices representing parasympathetic and overall autonomic control (standard deviation of the RR intervals, root mean square of successive differences between adjacent normal RR intervals, number of adjacent normal RR intervals differing by >50 ms, proportion of adjacent normal RR intervals differing by >50 ms, HF component, SD1, and SD2) presented positive correlations with spirometric indices. Those representing sympathetic modulation (the LF component and LF/HF ratio) presented negative correlations with those indices. The nonlinear index α2 correlated significantly with FVC. After multiple regression analysis, adjusted for the main confounders, only SD2 of the Poincaré plot and dyslipidemia remained as determinants of the spirometric indices (Table 3).

**Table:**
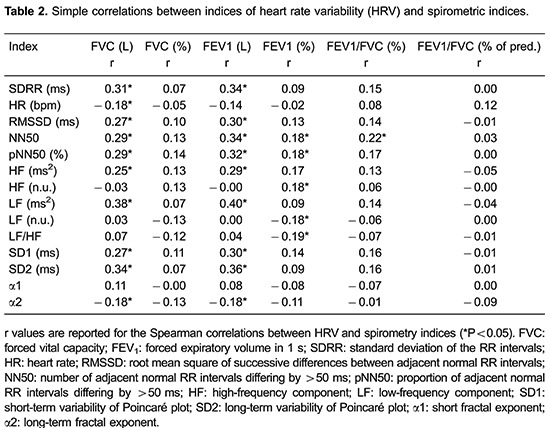

**Table:**
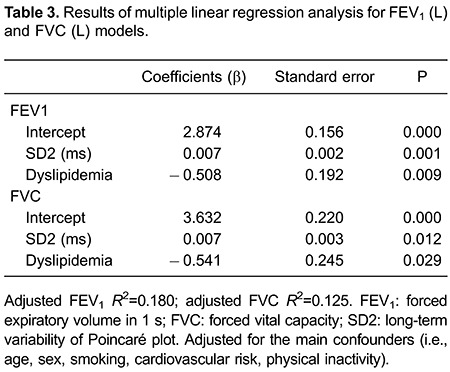

## Discussion

Here, we have demonstrated that pulmonary function correlated significantly with several HRV indices in asymptomatic adults. Although moderate, those correlations remained significant regardless of cardiovascular risk. Among cardiovascular risk factors, dyslipidemia was found to be the most important predictor of pulmonary function.

After adjusting for the main confounders, we found that the SD2 of the Poincaré plot remained as a determinant of FVC and FEV_1_. SD2 has been associated with overall HRV (1 2 3). Therefore, higher SD2 values indicate the predominance of the parasympathetic nervous system in autonomic balance. Although there have been few studies evaluating the Poincaré plot in healthy participants at rest, the evidence suggests that an increase in SD1 indicates increased parasympathetic activity, whereas an increase in SD2 indicates decreased sympathetic activity (1 2 3). Given that FEV_1_ has been shown to be an important risk factor for mortality and respiratory diseases (30) and that FVC is considered a measure of pulmonary capacity (30), our finding that autonomic control presented significant positive correlations with spirometric indices suggests that there is synchronicity between the activities of the lung and those of the heart. The predominance of the parasympathetic nervous system might be related to respiratory efficiency. That finding is in agreement with those of Hayano et al. (31), who showed that the HF component is a measure of parasympathetic nervous system activity. In the cardiopulmonary system, the HF component is an intrinsic resting function aimed at optimizing pulmonary function. It has also been suggested that the HF component creates functional synchrony between the heart and lungs by matching the timing of alveolar ventilation to capillary perfusion during the respiratory cycle, increasing the rate of gas exchange (32). However, even though the HF component is considered a valid index of parasympathetic nervous system input, the respiratory rate and tidal volume might be confounders of the association between the HF component and cardiac vagal tone (7). At rest (when the cardiac vagal tone is constant), decreased tidal volume and increased respiratory rate can attenuate the HF component (8). Our results show that, in addition to the HF component, SD2 is also related to synchronization of cardiac and pulmonary function, being representative of the autonomic balance. Rapid breathing is known to greatly attenuate the HF component (32), which could explain why we found that the HF component did not correlate with the spirometric indices when we used the FVC maneuver. Despite the modest *R* ^2^ values, our results suggest that autonomic control has an independent effect on pulmonary function in adults.

To date, there have been few studies investigating the correlations evaluated in the present study. To our knowledge, only one study has addressed such correlations in healthy adults; Behera et al. (23) also found that pulmonary function presented positive bivariate correlations with the parasympathetic and overall HRV indices, the HF component and FEV_1_, and negative correlations with the sympathetic HRV indices, the LF component and peak expiratory flow in healthy adults. After linear regression analysis, the authors observed a significant correlation between the HF component and FEV_1_/FVC ratio, indicating that HRV is a determinant of pulmonary function. However, only the frequency domain of HRV was used and the regression analysis was not adjusted for the main confounders, such as physical inactivity and other cardiovascular risk factors. In contrast, after adjusting for cardiovascular risk factors, we found that the frequency domain of HRV was not an important determinant of pulmonary function.

In the present study, multiple regression analysis revealed that dyslipidemia was also a determinant of pulmonary function (FEV_1_ and FVC). The important role of cholesterol as an inflammatory regulator might partially explain the relationship between pulmonary function and dyslipidemia. In addition, there is a correlation between total cholesterol and mortality from respiratory disease (15). Furthermore, dyslipidemia has been shown to correlate negatively with FEV_1_ (33). Our results underscore previous data indicating an inverse relationship between dyslipidemia and pulmonary function. That relationship might also be explained by the role of low density lipoprotein as an optimizer of inflammation, which, in conjunction with oxidative stress, increases the severity of pulmonary diseases (34). Age also plays an important role in the association between dyslipidemia and pulmonary function because aging individuals tend to show declines in FEV_1_ and FVC, as well as increased dyslipidemia (35). However, in the present study, we found that dyslipidemia was predictive of pulmonary function, even after adjusting for age. Therefore, it is reasonable to assert that dyslipidemia could have deleterious effects on lung tissue, affecting spirometric indices independently of other factors.

The association between pulmonary function and HRV was also independent of smoking, physical inactivity, and other cardiovascular risk factors. The influence of those factors on pulmonary function has previously been described (16,18,19). Among such factors, special attention should be given to physical activity in daily life, measured directly as in the present study. In a recent prospective study, an increase in physical activity level was found to prevent a decline in FVC among adolescents and young adults (36). In a 5-year cohort study (37), FEV_1_ was shown to increase by 50 mL in participants who remained active, whereas it declined by 40 mL in those who remained inactive. In an epidemiological study (38), a relatively large proportion of never smokers were found to have COPD (38). Certainly, there are other modifiable genetic or environmental risk factors that determine individual susceptibility (39). Although a low level of physical activity in daily life has been described as a consequence of COPD, recent studies raise the possibility that inactivity is actually a risk factor for the development and progression of the disease. It is plausible to suggest that a low level of physical activity in daily life has negative repercussions for pulmonary function because it increases oxidative stress and inflammation, which are commonly observed in sedentary individuals (40). In the present study, we observed an influence of HRV on FEV_1_ and FVC, independent of the well-established association between daily physical activity and pulmonary function. Therefore, our results suggest a complex interaction among cardiovascular risk factors, autonomic balance, and pulmonary function. Future studies should investigate these relationships in a longitudinal manner.

The present study has certain limitations. Because this was a cross-sectional study, we cannot know whether improvement of the HRV indices would prevent a decline in pulmonary function over time. In addition, we assessed cardiovascular risk factors through interviews, which could have led us to underestimate the influence of factors such as hypertension and diabetes on pulmonary function. However, the previously described interaction among inflammation, autonomic control, smoking, and physical inactivity supports our results. Furthermore, the correlations we observed between pulmonary function and autonomic control in adults free of cardiorespiratory disease have clinical relevance and should be considered when assessing the risk of respiratory diseases.

We conclude that pulmonary function is positively associated with autonomic control in asymptomatic adults, regardless of the confounding effects of cardiovascular risk factors. Among these factors, dyslipidemia seems to play an important role in determining pulmonary function. Our results suggest that increased parasympathetic activity is related to increased respiratory efficiency, whereas dyslipidemia is related to decreased pulmonary function. Therefore, strategies for improving autonomic control and reducing the impact of dyslipidemia in asymptomatic adults should be investigated in cohort studies, which might help prevent a decline in pulmonary function over time. Our results highlight the importance of the integrity of autonomic control to pulmonary function in asymptomatic adults.
